# Male Sexual Function after Allogeneic Hematopoietic Stem Cell Transplantation in Childhood: A Multicenter Study

**DOI:** 10.3390/cancers12071786

**Published:** 2020-07-04

**Authors:** Anu Haavisto, Sidsel Mathiesen, Anu Suominen, Päivi Lähteenmäki, Kaspar Sørensen, Marianne Ifversen, Anders Juul, Malene Mejdahl Nielsen, Klaus Müller, Kirsi Jahnukainen

**Affiliations:** 1 Department of Psychology and Logopedics, University of Helsinki, P.O. Box 21, 00014 Helsinki, Finland; anu.haavisto@helsinki.fi; 2 Department of Pediatrics and Adolescent Medicine, Rigshospitalet, University of Copenhagen, 2100 Copenhagen, Denmark; sidsel.mathiesen@regionh.dk (S.M.); kaspar.soerensen.01@regionh.dk (K.S.); marianne.ifversen@regionh.dk (M.I.); malene.mejdahl.nielsen.01@regionh.dk (M.M.N.); klaus.mueller@regionh.dk (K.M.); 3 Division of Haematology-Oncology and Stem Cell Transplantation, New Children’s Hospital, Pediatric Research Center, University of Helsinki and Helsinki University Hospital, 00029 Helsinki, Finland; anu.suominen@fimnet.fi; 4 Department of Pediatrics and Adolescent Medicine, Turku University Hospital and Turku University, 20520 Turku, Finland; paivi.maria.lahteenmaki@tyks.fi; 5 Department of Growth and Reproduction, Rigshospitalet, University of Copenhagen, 2100 Copenhagen, Denmark; anders.juul@regionh.dk; 6 Institute for Inflammation Research, Rigshospitalet, University of Copenhagen, 2100 Copenhagen, Denmark; 7 NORDFERTIL Research Lab Stockholm, Department of Women’s and Children’s Health, Karolinska Institutet and University Hospital, 17164 Stockholm, Sweden

**Keywords:** HSCT, children, long-term survivors, pediatric cancer, sexual dysfunction, late effects

## Abstract

There are many known endocrine complications after allogeneic hematopoietic stem cell transplantation (HSCT) in childhood including increased risk of biochemical hypogonadism. However, little is known about sexuality in adulthood following childhood HSCT. In this multicenter study, sexual functions and possible risk factors were assessed comprehensively in two national cohorts (Finland and Denmark) of male adult survivors of childhood HSCT. Compared to a healthy control group (*n* = 56), HSCT survivors (*n* = 97) reported less sexual fantasies, poorer orgasms, lower sexual activity with a partner and reduced satisfaction with their sex life, even in the presence of normal erectile functions and a similar frequency of autoerotic acts. Of the HSCT survivors, 35% were cohabitating/married and 66% were sexually active. Risk factors for poorer self-reported sexual functions were partner status (not cohabitating with a partner), depressive symptoms, CNS and testicular irradiation. Sexual dysfunction increased by age in the HSCT group with a pace comparable to that of the control group. However, because of the lower baseline level of sexual functions in the HSCT group, they will reach the level of clinically significant dysfunction at a younger age. Hence, male survivors of childhood HSCT should be interviewed in detail about their sexual health beyond erectile functions.

## 1. Introduction

Allogeneic hematopoietic stem cell transplantation (HSCT) is an established therapeutic procedure for a number of malignant and non-malignant hematological disorders. Despite the great improvements in clinical practices in recent decades, nearly all children treated with HSCT will experience one or more treatment-related late effects [1]. Those most frequently observed are endocrine complications such as hypogonadism, impaired fertility, growth delay and metabolic disturbances [2]. However, there is very little knowledge about sexual health in adulthood.

In adult survivors of childhood HSCT, sexual functions have previously only been reported on a general level as one of several aspects of quality of life. Pediatric HSCT survivors have rated family and relationships with a partner among the top 10 important areas in life [3]. However, compared to their peers, an increased proportion of these survivors are living with their parents and have never been married or cohabitated with a partner [4]. One study identified sexual functions and partner relations among the most affected areas of long-term quality of life [5]. 

In adult male HSCT recipients, an initial decline in sexual activity and sexual functions have been reported after HSCT with the majority experiencing recovery by 1–2 years post-HSCT. However, even five years after HSCT, recipients continued to report significantly lower sexual functions compared to peers [6]. Problems with erection, ejaculation and orgasm, pain during intercourse, diminished sexual interest and anxiety about sexual performance dominate [6,7,8]. 

Male cancer survivors, with or without HSCT, generally tend to participate less in studies and also report less psychological and sexual dysfunction compared to females [4,9,10]. In this national cohort study of two Nordic countries (Denmark and Finland), we therefore focused on a systematic evaluation of sexual functions in male adult survivors of childhood HSCT and associations to sociodemographic factors, therapy exposures, physical and psychological health.

## 2. Results

### 2.1. Clinical Data and Sex Hormones

At the time of HSCT, 71 patients were prepubertal, 16 pubertal and 9 post-pubertal (data not known for one patient). At the time of this study, all were post-pubertal (Tanner stages 4–5). Genital chronic graft-versus-host disease (cGvHD) was found in 16 patients; 11 were operated for phimosis that emerged after HSCT, 1 was operated for urethral stenosis that emerged after HSCT, 2 had lichen sclerosis, 1 had lichen planus like changes and was operated for phimosis that emerged after HSCT and 1 had a maculopapular rash on scrotum. Compared to the control group, the HSCT group had significant testicular damage, indicated by lower testosterone levels, lower testicular volumes and lower total sperm counts and more socioeconomic disadvantage, i.e., living without a partner, lower education and more unemployment (Table 1). 

### 2.2. Sexual Functioning

Of the 97 HSCT survivors, 63 (66%) reported to be sexually active (data not known for one patient). Of the 63 survivors not cohabitating with a partner, 29 (47%) were sexually active, while the percentage was 100% for the 34 men cohabitating with a partner. These data were not known for the control group. 

The HSCT and control groups were compared in a MANCOVA test regarding self-reported sexual functioning. After adjustment for age at assessment and employment status, a significant main effect of group was found for overall sexual functioning; F(5, 143) = 3.34, *p* = 0.007, Wilk’s Λ = 0.895, η_p_^2^ = 0.105. In Bonferroni-corrected univariate analyses, the HSCT group reported poorer functioning in all domains except sexual arousal (Table 2).

**Figure 1:**
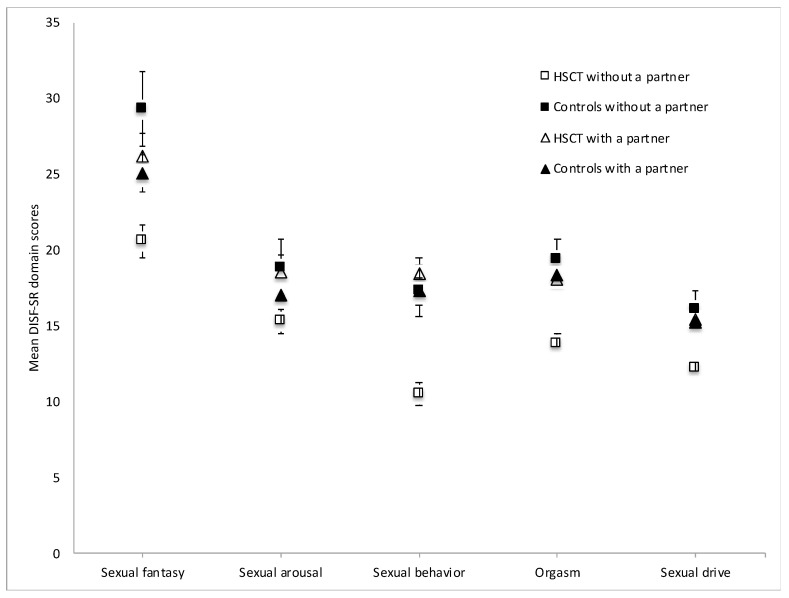
Mean scores on the Derogatis Interview for Sexual Functioning self-report (DISF-SR) in HSCT survivors and their controls categorized by partner status.

The MANCOVA also revealed a significant interaction between group and cohabitation status; F(5, 143) = 3.54, *p* = 0.005, Wilk’s Λ = 0.890, η_p_^2^ = 0.110. Bonferroni-corrected pairwise comparisons indicated that all men cohabitating with a partner, irrespective of group, reported similar sexual functioning. However, HSCT survivors not cohabitating with a partner had significantly poorer sexual functions compared to controls not cohabitating, regarding sexual fantasy (*p* = 0.002), sexual behavior (*p* < 0.001), orgasm (*p* < 0.001) and sexual drive (*p* = 0.004) (Figure 1). 

Concerning covariates included in the MANCOVA, there was no main effect of employment status on sexual functions. However, there was a main effect of age, with older men reporting poorer sexual functions across HSCT and control groups; F(5, 143) = 4.66, *p* = 0.001, Wilk’s Λ = 0.860, η_p_^2^ = 0.140. The effect was significant for sexual arousal (*p* = 0.015), orgasms (*p* < 0.001) and drive (*p* = 0.017). 

**Figure 2:**
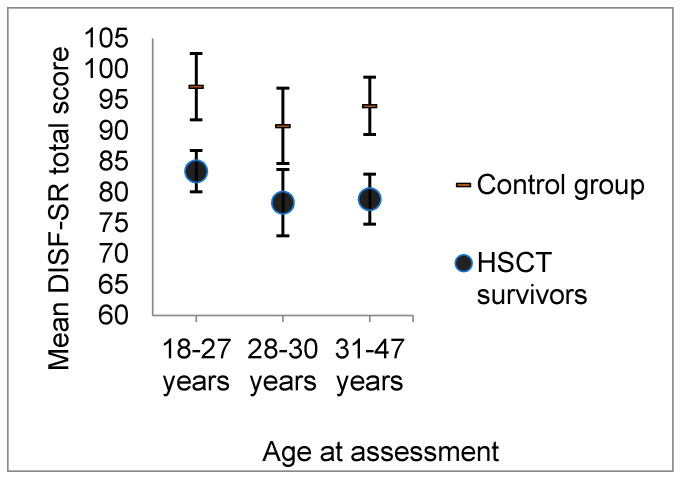
Mean total scores on the Derogatis Interview for Sexual Functioning self-report (DISF-SR) in HSCT survivors and their controls. Results are categorized according to age group. Sexual functions declined in a similar pace in both groups.

To analyze the effect of age between the HSCT and control groups, an age category (18–27, 28–30 and 31–47 years) was added as an independent variable in an additional MANCOVA. No group × age interaction emerged, indicating that sexual functions decreased with a similar pace in both groups; F(10, 272) = 0.375, *p* = 0.957, Wilk’s Λ = 0.973, η_p_^2^ = 0.014 (Figure 2).

### 2.3. Risk Factors for Reduced Sexual Functions within the HSCT Group

Regression analyses were carried out only within the HSCT group. Overall, self-reported depressive symptoms and CNS irradiation exposure were the single most important risk factors for poorer sexual functions (Table 3). Leydig cell function was associated with sexual cognition with the poorest scores observed in the survivors with ongoing testosterone substitution and the highest scores in those with normal serum testosterone levels (≥10 nmol/L). Similarly azoospermia correlated with poorer sexual function but since testosterone substitution affects spermatogenesis, azoospermia as an independent predictor was excluded from further analyses.

The treatment variables CNS and testicular irradiation made the model unstable due to their high multicollinearity. To analyze the effect of the multicollinearity, CNS and testicular irradiation exposure doses were included in the regression model one by one together with the other independent variables. These analyses showed that the multicollinearity affected the domain of sexual arousal and orgasm; cumulative doses of CNS and testicular irradiation replaced each other if only one of them was included in the analyses. 

## 3. Discussion

This is the first study using a standardized questionnaire to assess multiple domains of sexual functions in adult male survivors of childhood HSCT. Several domains of sexuality were affected. HSCT survivors had less sexual fantasies, engaged less in sexual activity together with a partner, had poorer orgasms, experienced erectile problems during intercourse and were less satisfied with their personal relationships compared to age-matched peers. However, survivors had a similar frequency of autoerotic acts and were capable of a full erection during these activities, indicating an interrelational rather than merely a physiological problem. 

Relationship status was strongly associated with sexual dysfunction. In line with previous studies, only 35% of the survivors were married or cohabitating [3,4]; a concern raised also among long-term survivors of childhood cancers treated without HSCT [11,12]. Moreover, half of the HSCT recipients living alone reported not being sexually active. In adult cancer survivors, the presence of a partner and a better post-treatment recovery of sexual functions are associated [13]. Hence, finding a partner is an important psychosexual milestone for these young survivors.

In a previous study of adult HSCT recipients, 25% of men reported erectile dysfunction often or always [7]. In the present study, the recipients of pediatric HSCT reported full erection during sexual fantasies and autoerotic acts, yet erectile dysfunction during intercourse and less frequent sexual activity with a sexual partner. This discrepancy has also been reported in conventionally treated adult male survivors of childhood cancer [11]. Our data indicate that for survivors of pediatric HSCT, sexual functions with a partner are particularly affected, even in the presence of normal erectile function. This observation supports the notion that problems with partner relationships are a major obstacle in decreased sexual functioning after pediatric HSCT.

Higher cumulative doses of radiotherapy exposing the CNS or testicles had an effect on the physical quality of arousal and orgasms, while sexual behavior was affected more by CNS irradiation. Radiotherapy exposing the CNS has an adverse effect on the hypothalamic–pituitary–gonadal axis, thyroid function and long-term sequelae on cognition. This may potentially affect sexual desire and the ability to form partnerships through hormonal abnormalities, short stature and cognitive impairment [14]. Similarly, radiotherapy exposing gonads may cause hypogonadism leading to azoospermia, Leydig cell damage and decreased serum testosterone, as well as direct irradiation damage to the spinal cord, sympathetic nerves or pelvis leading to physical sexual dysfunction [15]. Indeed, ongoing testosterone substitution, as a sign of significant hypogonadism, was associated with lower sexual cognition. The reasons for this association could not be explored in this study, but we observed greater variations in testosterone levels in the substituted group, compared to the non-substituted men. For some of the men, the testosterone substitution may not have been optimal. Further, the psychological burden associated with infertility may explain some of the observed sexual dysfunction [16]. No association between cumulative doses of alkylating agents and sexual function was observed. This is in line with the evidence that alkylating agents do not cause overt testosterone deficiency in early adulthood and, hence, have no independent effect on sexual function [15]. 

Self-reported depressive symptoms were strongly associated with dissatisfaction with sexual functioning. Depression and anxiety have been related to sexual dysfunction in adult recipients of HSCT [8] as well as in the general population [17]. The effect of depression and sexual dysfunction is best understood as bidirectional, both conditions affecting each other. 

Physical consequences such as endurance, measured by an objective test of walking distance over two minutes, or cGvHD, graded on the day of examination, were not associated with self-reported sexual functioning. Chronic GvHD has been associated with sexual dysfunction in some studies [18], while not in others [19]. However, adult recipients with genital cGvHD have reported more erectile dysfunction [20]. Genital cGvHD was rare in our patient sample, with no severe (Score 3) forms detected. Similarly, severe cGvHD was found in only 3%, while mild (Score 1) and moderate (Score 2) forms together were detected in almost one fourth of the sample. The present results suggest that psychosexual consequences, rather than physical functions alone, predict sexual dysfunction in this population. 

Sexual function declines with age in the general population, an effect also observed in this study. Older age at assessment was associated with more functional problems in the domains of arousal and orgasm as well as with sexual drive. The difference between the HSCT and control groups remained stable over time. Age or pubertal status at HSCT, educational level or employment status were not associated with sexual health in this study. In sum, age, but not other demographic risk factors, was important for long-term sexual outcome. Due to their lower initial level of sexual functions, the HSCT recipients are expected to reach the level of clinically significant dysfunction at a younger age than their healthy peers.

The consequences of HSCT on sexual functioning are not routinely discussed at health care visits. In adult HSCT recipients approximately half of the patients reported that their health care provider had discussed consequences on sexual functioning (excluding fertility) [21] while the number was as low as 38% in adult survivors of childhood HSCT [5]. Patients wish for the initiative for discussions on sexual matters to come from health care professionals [5]. The high response and participation rates in our study suggest that long-term survivors of pediatric HSCT are interested in their gonadal function. Since it may be uncomfortable for many patients to bring up sexual matters, it is important for health care providers to ask about genital skin symptoms, partners and sexual dysfunction. Clinicians need structured guidelines how to interview young survivors about their sexual health. 

A limitation of this study was that the control group was recruited from occupational health services, and all were employed. The employment rate of 88% in the HSCT survivors was similar to those reported by others (82–86%) [4,22]. Employment status was controlled for in the group comparisons and was not associated with sexual functions. Hence, it does not appear to have skewed the results. Further, the control group was collected 9 years prior to the patient group. An important extension of this study would be to assess the experiences of the survivors’ partners. This would offer a valuable point of view for studying the more well-functioning HSCT survivors in more detail. Our response (70%) and participation rates (54%) were good considering the extensive evaluations to which the participants agreed. 

## 4. Materials and Methods

This study is part of a larger cross-sectional cohort study addressing long-term gonadal function, metabolism, immune function, physical fitness, sexual health and quality of life. It included two national cohorts of men who before the age of 17 years had undergone HSCT in Denmark or Finland between 1980 and 2010 and who were at least 18 years by 1st of January 2017. Patients were excluded from this study if they were unable to give informed consent, if language difficulties compromised the examinations or if patients had undergone more than one HSCT. In the Finnish cohort, all eligible patients were contacted by mail. In the Danish cohort, this was carried out by mail and phone. 

A total of 181 eligible patients were identified—of which 55 could not be reached. Of the 126 patients who were contacted, 5 were excluded because they were unable to give informed consent (4 due to intellectual disability and 1 due to language difficulties) and 24 declined participation in this substudy. Thus, 97 survivors (49 Danish and 48 Finnish) participated in this study, giving a participation rate of 54%. Altogether, 59 (60%) of the participants had a malignant diagnosis. There were no statistically significant differences in the numbers of malignancies, age at HSCT, follow-up time or other transplant characteristics between participants and non-participants [23] (data not shown). The examination consisted of an updated medical anamnesis, questionnaires, laboratory measurements and a physical examination. All examinations were carried out between January and October 2017.

An age- and gender-matched control group was recruited from the occupational health services of the Helsinki municipality area and the Helsinki University Central Hospital. A total of 56 men without a history of cancer or HSCT participated. They underwent the same examinations between August 2005 and June 2008.

### 4.1. Self-Report Measure of Sexual Functioning

Sexual functioning was assessed by the Derogatis Interview for Sexual Functioning self-report (DISF-SR) [24]. It comprises 25 questions which form five domains: sexual cognition/fantasy (5 questions on the frequency of sexual thoughts and fantasies), sexual arousal (5 questions on the frequency of a full erection during, e.g., sexual fantasies, masturbation and sex), sexual behavior/experience (5 questions on the frequency of sexual acts such as engaging in books/films with erotic content, masturbation, foreplay and intercourse), orgasm (6 questions on how content the respondent is with his orgasms) and sexual drive/relationship (4 questions about the ideal frequency of sexual intercourse, interest in sex, satisfaction with the current sexual relationship and quality of sexual functioning). The questions were rated on a five- or nine-point Likert scale. Total scores were calculated for each domain as well as a global summary score (total score). Higher scores indicate better functioning and a higher sexual activity level.

### 4.2. Risk Factors

Data on relationship status and sexual activity were collected during a structured interview, while other background and social variables were collected by questionnaire. Self-reported depressive symptoms were evaluated with the Beck Depression Inventory (BDI-21) [25]. It comprises 21 questions rated on a four-point Likert scale. Lower scores indicate fewer depressive symptoms. Since question number 19 about weight loss was different in the Danish and Finnish versions, it was removed. The two-minute walk test was used as an objective measure of physical fitness and a z-score was calculated [26]. 

The survivors’ medical history was reviewed from medical records including therapeutic pre-HSCT exposure and exposure at conditioning to irradiation (testicular, CNS and total body irradiation, cGy) and alkylating agents. Alkylating agents were converted into a cumulative cyclophosphamide equivalent dose (CED, mg/m^2^) [27]. 

Testicular volume and hormone levels were assessed on the day of examination. Blood samples were taken before 10 a.m. and analyzed in the laboratories of the Helsinki University Hospital and Rigshospitalet according to the hospitals’ routine methods. Free testosterone was calculated from total testosterone and SHBG, with a fixed albumin level of 43 × 8 g/L [28]. Testicular volume was measured by an orchidometer (mL). Mean volume of the testicles was calculated, when applicable. Chronic GvHD was assessed by examination according to the National Institutes of Health criteria [29]. Previous phimosis or urethral stenosis that emerged after transplantation, but was later surgically removed, was considered a diagnostic sign of cGvHD with genital symptom score 1. The methods have been described in more detail in Mathiesen et al. [23].

### 4.3. Statistical Analyses

Statistical calculations were carried out using IBM SPSS Statistics 24.0. Group comparisons of the background variables were undertaken with independent samples *t* test, Mann–Whitney U test and Fisher’s exact χ^2^ test.

Sexual functioning was analyzed in a MANCOVA, with the five DISF-SR domain scores as the dependent variables. The domain scores were normally distributed by visual inspection. The independent variables were group (HSCT and control group) and cohabitating with a partner (yes/no). Adjustments were made for two covariates: age at assessment and employment status (full- or part time work or studies, yes/no). Employment status was controlled for because it was significantly different between the groups. Bonferroni correction was used for post-hoc tests. Additionally, the DISF-SR questions were analyzed on item level using independent-samples t tests corrected for multiple comparisons with the false discovery rate.

To assess the effect of age, an additional MANCOVA was undertaken with the DISF-SR domain scores as dependent variables. The independent variables were group (HSCT vs. control group), cohabitating with a partner (yes/no) and age category (three classes). The age categories were 18–27 (*n* = 47), 28–30 (*n* = 18) and 31–47 years (*n* = 32). The analysis was adjusted for employment status.

Risk factors were analyzed in forward multiple regression analyses with the DISF-SR domain scores used as dependent variables in separate analyses. The risk factors included as independent variables were related to background variables (age at assessment, educational level), treatment (age at HSCT, CED, total cumulative radiotherapy exposing the testicles and CNS) and physiological, physical and psychological functioning at the time of assessment (Leydig cell function, cGvHD, two-minute walk test z-score, BDI-21 score). Three testosterone groups were defined based on Leydig cell function: (1) ongoing testosterone substitution, (2) low serum testosterone level (<10 nmol/L) and (3) normal testosterone level (≥10 nmol/L). The mean (SD) testosterone values for the groups were: (1) 20.3 (12.1), (2) 6.6 (2.5) and (3) 17.4 (5.2) nmol/L. Age at assessment and follow-up time were significantly correlated and only age was included in the regression model. Increased TSH levels correlated with poorer sexual behavior and drive, but the need for thyroxin substitution had a strong intercorrelation with CNS irradiation. Hence, TSH levels could not be included in the regression analyses. Tanner stage at HSCT, total sperm count, free testosterone, SHBG and prolactin were not correlated with the DISF-SR and thus not included.

In the HSCT group, there were a total of 14 missing item responses in the DISF-II domain of sexual drive (3.6% missing data in the domain) in eight patients. Seven of these were sexually inactive and had not rated their satisfaction with the current sexual relationship. Since most men with inactive sex lives in this study group had rated this question with the lowest possibly score of 0, these seven missing replies were given a score of 0. The rest of the missing data (seven item responses) were estimated using the expectation-maximization algorithm. All tests of significance were two sided (*p* < 0.05). η_p_^2^ and *R*^2^ served as indicators of effect size.

### 4.4. Ethics

This study was approved by the Regional Committee on Health Research Ethics, Denmark (H-16043059), the Danish Data Protection Agency (RH-2017-58), the Helsinki University Hospital (HUS/1742/2016) and the Research Ethics Committee of the Helsinki University Hospital (DNR no. 1905-32/300/05). All participants provided written informed consent.

## 5. Conclusions

In conclusion, a standardized questionnaire assessing multiple domains of sexual functions identified lower sexual activity and poorer orgasms in adult male survivors of childhood HSCT. Sexual functions with a partner were affected even in the presence of normal erectile function. Sexual dysfunction was strongly associated with the presence of depressive symptoms, the absence of a life partner, and cumulative doses of radiotherapy exposing the CNS and the testicles with resulting azoospermia and reduced testosterone production, but not with cGvHD or physical endurance. Our results suggest that psychosexual consequences, rather than physical deficiencies, predict sexual dysfunction in this population. Male survivors without a partner, in particular, should be interviewed in detail about their sexual health beyond erectile functions.

## Figures and Tables

**Table 1:** Background and laboratory characteristics in pediatric hematopoietic stem cell transplantation (HSCT) survivors and controls.

Characteristic	HSCT (*n* = 97)	Range	Controls (*n* = 56)	Range	*p*-Value
Background variables	Mean (SD)		Mean (SD)		
Age at assessment	28.8 (7.3)	18.5–47.0	29.6 (3.2)	24.0–36.0	0.334
Age at diagnosis	8.7 (4.4)	0.2–16.4			
Age at HSCT	9.8 (4.2)	0.4–16.9			
Follow-up time since HSCT	19.0 (7.0)	7.7–34.6			
Diagnostic groups	*n* (%)				
ALL	45 (46%)				
NHL	5 (5%)				
AML	9 (9%)				
SAA	14 (14%)				
Other	24 (25%)				
Social and demographic variables	*n* (%)		*n* (%)		
Married or cohabitating	34 (35%)		45 (80%)		<0.001
Biological children	6 (6%)		24 (43%)		<0.001
*Highest education*					<0.001
Comprehensive school (9 years)	20 (21%)		3 (5%)		
Matriculation examination or vocational school (12 years)	50 (52%)		20 (36%)		
Lower tertiary education (15 years)	13 (13%)		23 (41%)		
Higher tertiary education (min. 17 years)	14 (14%)		10 (18%)		
*Employment*					0.009
Employed/student (full or part time)	85 (88%)		56 (100%)		
Treatment regimens	*n* (%)				
Cancer treatment prior to HSCT	65 (67%)				
Total body irradiation	71 (73%)				
	Median				
Cumulative gonadal irradiation dose (cGy)	1000	0–4130			
Cumulative CNS irradiation dose (cGy)	1000	0–3600			
Cumulative cyclophosphamide equivalent doses (mg/m^2^) ^1^	5113	0–28,743			
Laboratory variables	Median		Median		
Inhibin B (ng/L) ^3^	21.0	1.0–247.0	157.0	74.0–307.0	<0.001
FSH (IU/L)	9.8	0.01–64.4	3.2	0.9–11.4	<0.001
LH (IU/L)	5.0	0.01–31.0			
SHBG (nmol/L) ^1^	26.4	4.8–68.8	32.5	13.0–70.0	0.004
Prolactin (mU/L)	209	44–888	216	101–634	0.350
TSH (mU/L)	2.5	0.03–9.8			
Total testosterone (nmol/L)	15.4	1.7–53.8	18.4	7.1–32.6	0.010
Free testosterone (pmol/L) ^1^	350	38–1470	296.5	151–406	0.002
Mean testicular volume (mL) ^2^	13.5	2.0–27.0	33.9	22.2–63.9	<0.001
Total sperm count, millions ^4^	0.00	0.00–524.8	135.5	17.0–562.0	<0.001
Treatment-related variables	*n* (%)				
Azoospermia, *n* (%) ^5^	61 (67%)		0		<0.001
Ongoing testosterone substitution	24 (25%)		0		<0.001
Ongoing thyroxin substitution	25 (26%)				
Chronic GvHD at the time of assessment	27 (28%)				
Grade 1	13 (13%)				
Grade 2	11 (11%)				
Grade 3	3 (3%)				

Abbreviations: ALL, acute lymphoblastic leukemia; AML, acute myeloid leukemia; CNS, central nervous system; FSH, follicle-stimulating hormone; GvHD, graft-versus-host disease; HSCT, hematopoietic stem cell transplantation; LH, luteinizing hormone; SHBG, sex hormone-binding globulin; TSH, thyrotropin; NHL, non-Hodgkin lymphoma; SAA, severe aplastic anemia. Data are presented as the mean (SD) and range with *p*-values from independent samples t test, median and range with *p*-values from independent samples Mann–Whitney U test, or *n* (%) with *p*-values from the exact χ^2^ test. ^1^ Data missing for one HSCT patient. ^2^ Data missing for four HSCT with bilateral orchiectomy, and four controls. ^3^ Data missing for four controls. ^4^ Data missing for six HSCT patients and two controls. ^5^ Data missing for six HSCT patients.

**Table 2:** Mean values (SD) in the Derogatis Interview for Sexual Functioning self-report between HSCT survivors and a healthy control group, with domain *p*-values from MANCOVA with Bonferroni-corrected post-hoc tests and item *p*-values from independent samples *t* tests corrected with the false discovery rate.

Domain Item	HSCT (*n* = 97)	Controls (*n* = 56)	*p*-Value
**Sexual fantasy (about)**	**22.6 (9.2)**	**25.8 (6.6)**	**0.023**
Attractive person	5.1 (2.0)	5.8 (1.6)	0.074
Erotic parts of a woman	5.0 (2.0)	5.5 (2.1)	0.675
Erotic situation	4.4 (2.0)	5.0 (1.6)	0.204
Foreplay	4.0 (2.2)	4.8 (1.8)	0.029
Intercourse	4.1 (2.3)	4.8 (1.6)	0.074
**Sexual arousal (Full erection)**	**16.5 (6.5)**	**17.3 (5.9)**	**0.423**
When waking up	3.2 (1.8)	3.4 (1.9)	1.00
During a fantasy	3.4 (2.0)	3.6 (2.1)	1.00
During a visual cue	3.8 (1.8)	3.4 (1.7)	0.651
During masturbation	3.8 (1.6)	3.5 (1.6)	0.342
During intercourse	2.3 (2.0)	3.2 (1.7)	0.002
**Sexual behavior**	**13.3 (7.0)**	**17.3 (4.7)**	**0.016**
Erotic books	2.6 (2.0)	2.0 (1.9)	0.310
Masturbation	3.7 (1.5)	3.2 (1.6)	0.216
Kissing and petting	3.0 (2.7)	5.4 (2.0)	<0.001
Foreplay	2.1 (2.0)	3.4 (1.8)	<0.001
Intercourse	2.0 (2.0)	3.3 (1.7)	<0.001
**Orgasm**	**15.3 (5.6)**	**18.6 (3.8)**	**0.002**
Ability	2.8 (1.1)	3.4 (0.9)	0.006
Intensity	2.7 (1.0)	3.2 (0.7)	0.002
Duration	2.5 (1.1)	3.0 (0.9)	0.006
Amount of seminal fluid	2.2 (1.2)	3.0 (0.9)	<0.001
Sense of control	2.3 (1.1)	2.7 (0.8)	0.078
Feeling of relaxation afterward	2.8 (1.0)	3.3 (0.9)	0.006
**Sexual drive**	**13.4 (4.4)**	**15.3 (3.4)**	**0.022**
Ideal frequency of intercourse	4.6 (1.7)	4.5 (0.8)	1.00
Interest in sex	2.4 (0.9)	2.8 (0.7)	0.075
Satisfaction with personal relationship	1.8 (1.5)	2.6 (1.1)	<0.001
Quality of current sexual functioning	4.5 (2.2)	5.5 (1.8)	0.012

Note. Scoring ranges for individual domains are: 0–40 for sexual fantasy, arousal and behavior; and 0–24 for orgasm and drive. Higher scores indicate better sexual functioning. Domain scores are indicated in bold.

**Table 3:** Multiple regression analyses of risk factors associated with self-reported sexual functions in the Derogatis Interview for Sexual Functioning self-report among HSCT survivors (*n* = 94).

Domain	Risk Factors	Standardized β	Adjusted R^2^
Sexual cognition/fantasy	BDI-21	−0.234 *	0.094
	Testosterone group	0.214 *	
Sexual arousal ^a^			0.146
	BDI-21	−0.289 **	
	CNS irradiation exposure	−0.215 *	
Sexual behavior/experience ^b^			0.043
	CNS irradiation exposure	−0.231 *	
Orgasm ^c^			0.368
	BDI-21	−0.431 ***	
	CNS irradiation exposure	−0.240 **	
	Age	−0.206 *	
Sexual drive/relationship			0.196
	BDI-21	−0.453 ***	

Abbreviations: BDI-21, Beck Depression Inventory; CNS, central nervous system. Note. The model included the following independent variables: age, education level, age at HSCT, cumulative cyclophosphamide equivalent dose, cumulative testicular and CNS irradiation exposure, testosterone group, cGvHD, two-minute walk test and BDI-21. In the DISF-SR, higher scores indicate better sexual functioning, while in BDI-21, lower scores indicate less depressive behavior. ^a^ When CNS irradiation was excluded from the analysis, BDI (β −0.326; *p* < 0.01) and testicular irradiation exposure (β −0.195, *p* < 0.05) had a significant association (adjusted R^2^ = 0.140). ^b^ When CNS irradiation was excluded from the analysis, no associations to risk factors emerged. ^c^ When CNS irradiation was excluded from the analysis, BDI (β −0.477; *p* < 0.001), age (β −0.198; *p* < 0.05) and testicular irradiation exposure (β −0.180, *p* < 0.05) had a significant association (adjusted R^2^ = 0.345). *** *p* < 0.001, ** *p* < 0.01, * *p* < 0.05.
